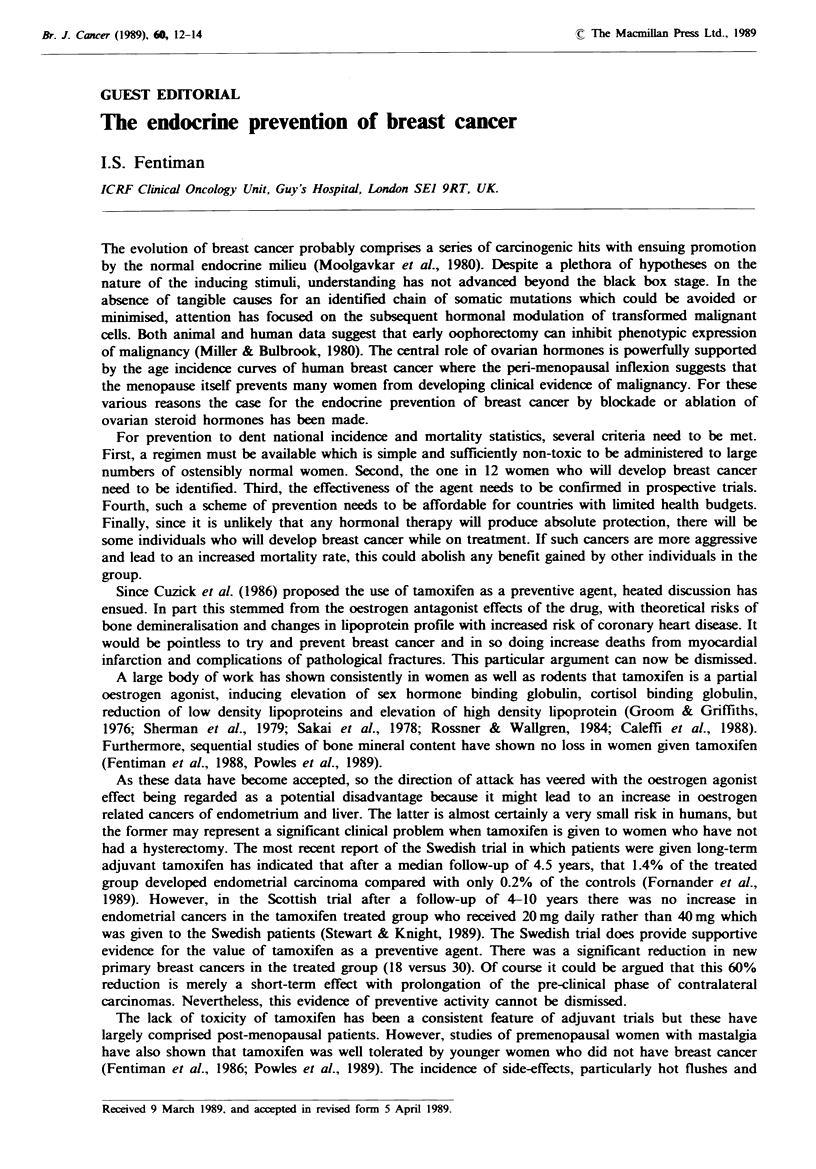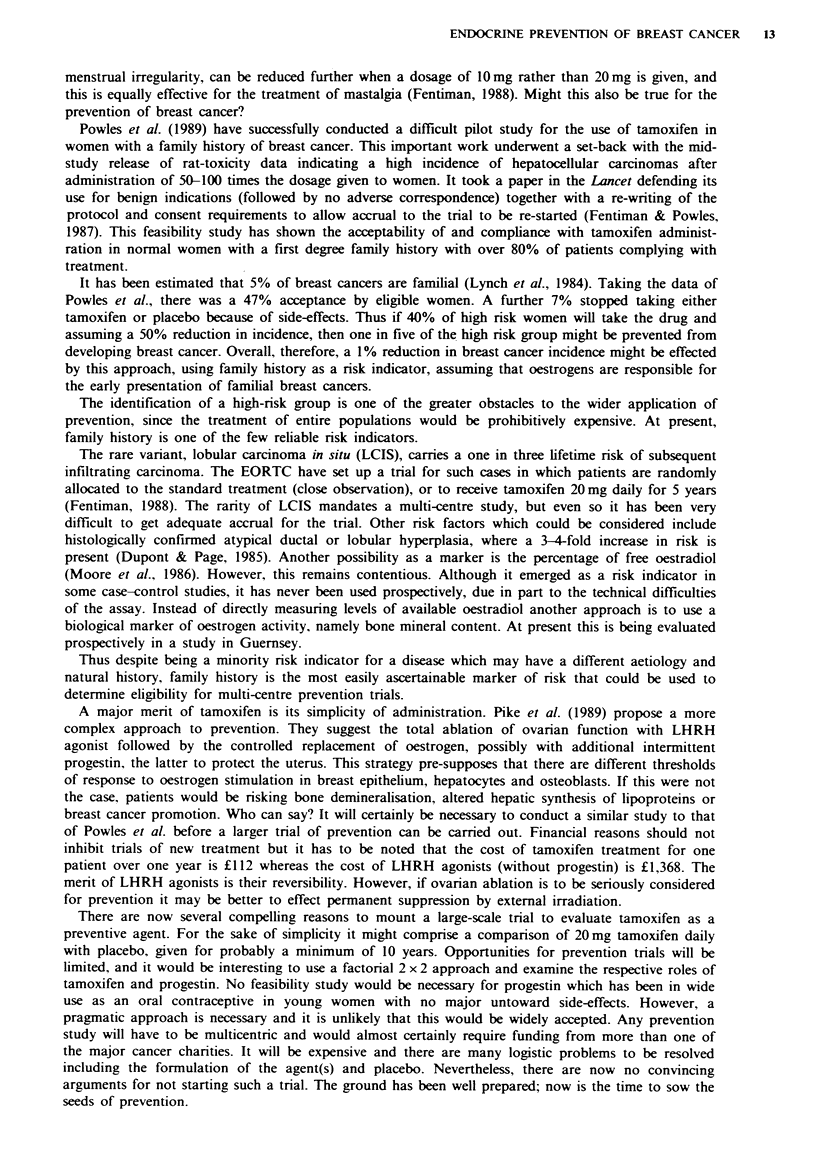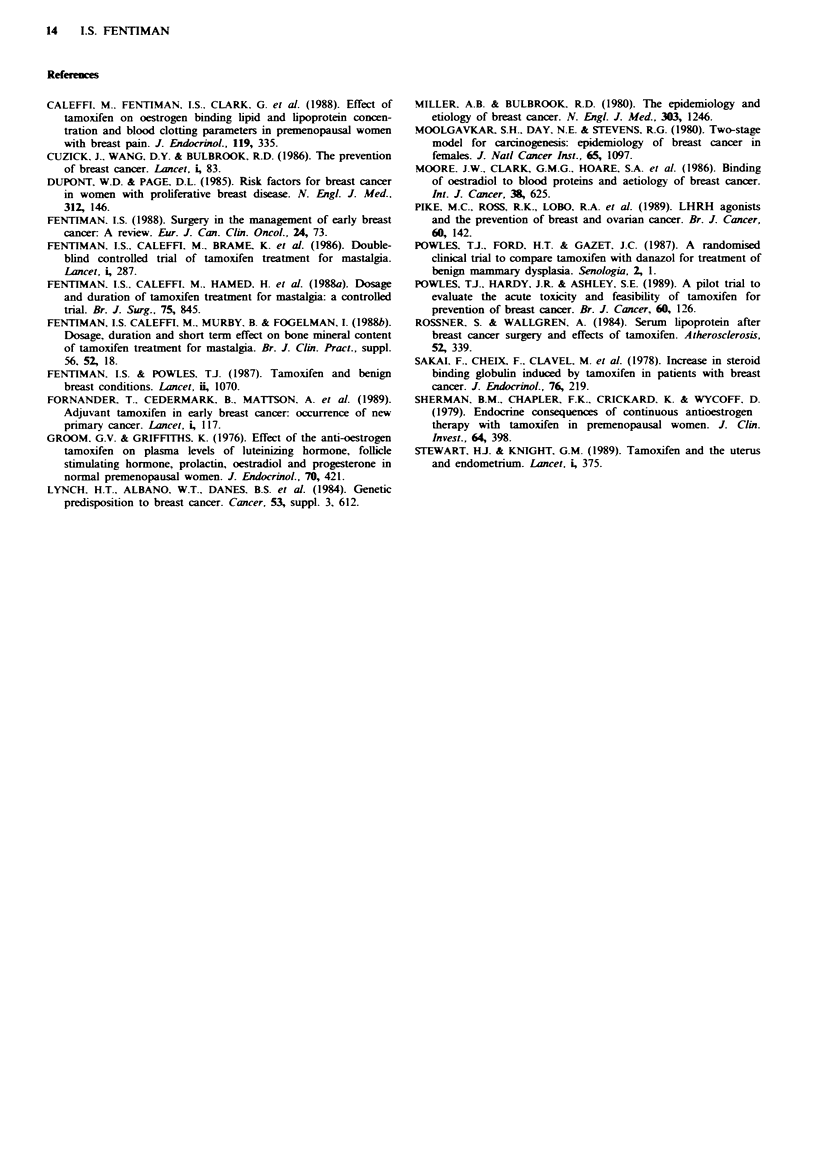# The endocrine prevention of breast cancer.

**DOI:** 10.1038/bjc.1989.210

**Published:** 1989-07

**Authors:** I. S. Fentiman

**Affiliations:** ICRF Clinical Oncology Unit, Guy's Hospital, London, UK.


*Br. J. Cancer* (1989), **60**, 12–14 © The Macmillan Press Ltd., 1989

GUEST EDITORIAL

### The endocrine prevention of breast cancer

I.S. Fentiman

*ICRF Clinical Oncology Unit, Guy's Hospital, London SE1 9RT, UK.*

The evolution of breast cancer probably comprises a series of carcinogenic hits with ensuing promotion by the normal endocrine milieu (Moolgavkar *et al.*, 1980). Despite a plethora of hypotheses on the nature of the inducing stimuli, understanding has not advanced beyond the black box stage. In the absence of tangible causes for an identified chain of somatic mutations which could be avoided or minimised, attention has focused on the subsequent hormonal modulation of transformed malignant cells. Both animal and human data suggest that early oophorectomy can inhibit phenotypic expression of malignancy (Miller & Bulbrook, 1980). The central role of ovarian hormones is powerfully supported by the age incidence curves of human breast cancer where the peri-menopausal inflexion suggests that the menopause itself prevents many women from developing clinical evidence of malignancy. For these various reasons the case for the endocrine prevention of breast cancer by blockade or ablation of ovarian steroid hormones has been made.

For prevention to dent national incidence and mortality statistics, several criteria need to be met. First, a regimen must be available which is simple and sufficiently non-toxic to be administered to large numbers of ostensibly normal women. Second, the one in 12 women who will develop breast cancer need to be identified. Third, the effectiveness of the agent needs to be confirmed in prospective trials. Fourth, such a scheme of prevention needs to be affordable for countries with limited health budgets. Finally, since it is unlikely that any hormonal therapy will produce absolute protection, there will be some individuals who will develop breast cancer while on treatment. If such cancers are more aggressive and lead to an increased mortality rate, this could abolish any benefit gained by other individuals in the group.

Since Cuzick *et al.* (1986) proposed the use of tamoxifen as a preventive agent, heated discussion has ensued. In part this stemmed from the oestrogen antagonist effects of the drug, with theoretical risks of bone demineralisation and changes in lipoprotein profile with increased risk of coronary heart disease. It would be pointless to try and prevent breast cancer and in so doing increase deaths from myocardial infarction and complications of pathological fractures. This particular argument can now be dismissed.

A large body of work has shown consistently in women as well as rodents that tamoxifen is a partial oestrogen agonist, inducing elevation of sex hormone binding globulin, cortisol binding globulin, reduction of low density lipoproteins and elevation of high density lipoprotein (Groom & Griffiths, 1976; Sherman *et al.*, 1979; Sakai *et al.*, 1978; Rossner & Wallgren, 1984; Caleffi *et al.*, 1988). Furthermore, sequential studies of bone mineral content have shown no loss in women given tamoxifen (Fentiman *et al.*, 1988, Powles *et al.*, 1989).

As these data have become accepted, so the direction of attack has veered with the oestrogen agonist effect being regarded as a potential disadvantage because it might lead to an increase in oestrogen related cancers of endometrium and liver. The latter is almost certainly a very small risk in humans, but the former may represent a significant clinical problem when tamoxifen is given to women who have not had a hysterectomy. The most recent report of the Swedish trial in which patients were given long-term adjuvant tamoxifen has indicated that after a median follow-up of 4.5 years, that 1.4% of the treated group developed endometrial carcinoma compared with only 0.2% of the controls (Fornander *et al.*, 1989). However, in the Scottish trial after a follow-up of 4–10 years there was no increase in endometrial cancers in the tamoxifen treated group who received 20 mg daily rather than 40 mg which was given to the Swedish patients (Stewart & Knight, 1989). The Swedish trial does provide supportive evidence for the value of tamoxifen as a preventive agent. There was a significant reduction in new primary breast cancers in the treated group (18 versus 30). Of course it could be argued that this 60% reduction is merely a short-term effect with prolongation of the pre-clinical phase of contralateral carcinomas. Nevertheless, this evidence of preventive activity cannot be dismissed.

The lack of toxicity of tamoxifen has been a consistent feature of adjuvant trials but these have largely comprised post-menopausal patients. However, studies of premenopausal women with mastalgia have also shown that tamoxifen was well tolerated by younger women who did not have breast cancer (Fentiman *et al.*, 1986; Powles *et al.*, 1989). The incidence of side-effects, particularly hot flushes and

Received 9 March 1989, and accepted in revised form 5 April 1989.

menstrual irregularity, can be reduced further when a dosage of 10 mg rather than 20 mg is given, and this is equally effective for the treatment of mastalgia (Fentiman, 1988). Might this also be true for the prevention of breast cancer?

Powles *et al.* (1989) have successfully conducted a difficult pilot study for the use of tamoxifen in women with a family history of breast cancer. This important work underwent a set-back with the mid-study release of rat-toxicity data indicating a high incidence of hepatocellular carcinomas after administration of 50–100 times the dosage given to women. It took a paper in the *Lancet* defending its use for benign indications (followed by no adverse correspondence) together with a re-writing of the protocol and consent requirements to allow accrual to the trial to be re-started (Fentiman & Powles, 1987). This feasibility study has shown the acceptability of and compliance with tamoxifen administration in normal women with a first degree family history with over 80% of patients complying with treatment.

It has been estimated that 5% of breast cancers are familial (Lynch *et al.*, 1984). Taking the data of Powles *et al.*, there was a 47% acceptance by eligible women. A further 7% stopped taking either tamoxifen or placebo because of side-effects. Thus if 40% of high risk women will take the drug and assuming a 50% reduction in incidence, then one in five of the high risk group might be prevented from developing breast cancer. Overall, therefore, a 1% reduction in breast cancer incidence might be effected by this approach, using family history as a risk indicator, assuming that oestrogens are responsible for the early presentation of familial breast cancers.

The identification of a high-risk group is one of the greater obstacles to the wider application of prevention, since the treatment of entire populations would be prohibitively expensive. At present, family history is one of the few reliable risk indicators.

The rare variant, lobular carcinoma *in situ* (LCIS), carries a one in three lifetime risk of subsequent infiltrating carcinoma. The EORTC have set up a trial for such cases in which patients are randomly allocated to the standard treatment (close observation), or to receive tamoxifen 20 mg daily for 5 years (Fentiman, 1988). The rarity of LCIS mandates a multi-centre study, but even so it has been very difficult to get adequate accrual for the trial. Other risk factors which could be considered include histologically confirmed atypical ductal or lobular hyperplasia, where a 3–4-fold increase in risk is present (Dupont & Page, 1985). Another possibility as a marker is the percentage of free oestradiol (Moore *et al.*, 1986). However, this remains contentious. Although it emerged as a risk indicator in some case–control studies, it has never been used prospectively, due in part to the technical difficulties of the assay. Instead of directly measuring levels of available oestradiol another approach is to use a biological marker of oestrogen activity, namely bone mineral content. At present this is being evaluated prospectively in a study in Guernsey.

Thus despite being a minority risk indicator for a disease which may have a different aetiology and natural history, family history is the most easily ascertainable marker of risk that could be used to determine eligibility for multi-centre prevention trials.

A major merit of tamoxifen is its simplicity of administration. Pike *et al.* (1989) propose a more complex approach to prevention. They suggest the total ablation of ovarian function with LHRH agonist followed by the controlled replacement of oestrogen, possibly with additional intermittent progestin, the latter to protect the uterus. This strategy pre-supposes that there are different thresholds of response to oestrogen stimulation in breast epithelium, hepatocytes and osteoblasts. If this were not the case, patients would be risking bone demineralisation, altered hepatic synthesis of lipoproteins or breast cancer promotion. Who can say? It will certainly be necessary to conduct a similar study to that of Powles *et al.* before a larger trial of prevention can be carried out. Financial reasons should not inhibit trials of new treatment but it has to be noted that the cost of tamoxifen treatment for one patient over one year is £112 whereas the cost of LHRH agonists (without progestin) is £1,368. The merit of LHRH agonists is their reversibility. However, if ovarian ablation is to be seriously considered for prevention it may be better to effect permanent suppression by external irradiation.

There are now several compelling reasons to mount a large-scale trial to evaluate tamoxifen as a preventive agent. For the sake of simplicity it might comprise a comparison of 20 mg tamoxifen daily with placebo, given for probably a minimum of 10 years. Opportunities for prevention trials will be limited, and it would be interesting to use a factorial $2 \times 2$ approach and examine the respective roles of tamoxifen and progestin. No feasibility study would be necessary for progestin which has been in wide use as an oral contraceptive in young women with no major untoward side-effects. However, a pragmatic approach is necessary and it is unlikely that this would be widely accepted. Any prevention study will have to be multicentric and would almost certainly require funding from more than one of the major cancer charities. It will be expensive and there are many logistic problems to be resolved including the formulation of the agent(s) and placebo. Nevertheless, there are now no convincing arguments for not starting such a trial. The ground has been well prepared; now is the time to sow the seeds of prevention.

**References**

CALEFFI, M., FENTIMAN, I.S., CLARK, G. *et al.* (1988). Effect of tamoxifen on oestrogen binding lipid and lipoprotein concentration and blood clotting parameters in premenopausal women with breast pain. *J. Endocrinol.*, **119**, 335.

CUZICK, J., WANG, D.Y. & BULBROOK, R.D. (1986). The prevention of breast cancer. *Lancet*, **i**, 83.

DUPONT, W.D. & PAGE, D.L. (1985). Risk factors for breast cancer in women with proliferative breast disease. *N. Engl. J. Med.*, **312**, 146.

FENTIMAN, I.S. (1988). Surgery in the management of early breast cancer: A review. *Eur. J. Can. Clin. Oncol.*, **24**, 73.

FENTIMAN, I.S., CALEFFI, M., BRAME, K. *et al.* (1986). Double-blind controlled trial of tamoxifen treatment for mastalgia. *Lancet*, **i**, 287.

FENTIMAN, I.S., CALEFFI, M., HAMED, H. *et al.* (1988*a*). Dosage and duration of tamoxifen treatment for mastalgia: a controlled trial. *Br. J. Surg.*, **75**, 845.

FENTIMAN, I.S. CALEFFI, M., MURBY, B. & FOGELMAN, I. (1988*b*). Dosage, duration and short term effect on bone mineral content of tamoxifen treatment for mastalgia. *Br. J. Clin. Pract.*, suppl. 56, **52**, 18.

FENTIMAN, I.S. & POWLES, T.J. (1987). Tamoxifen and benign breast conditions. *Lancet*, **ii**, 1070.

FORNANDER, T., CEDERMARK, B., MATTSON, A. *et al.* (1989). Adjuvant tamoxifen in early breast cancer: occurrence of new primary cancer. *Lancet*, **i**, 117.

GROOM, G.V. & GRIFFITHS, K. (1976). Effect of the anti-oestrogen tamoxifen on plasma levels of luteinizing hormone, follicle stimulating hormone, prolactin, oestradiol and progesterone in normal premenopausal women. *J. Endocrinol.*, **70**, 421.

LYNCH, H.T., ALBANO, W.T., DANES, B.S. *et al.* (1984). Genetic predisposition to breast cancer. *Cancer*, **53**, suppl. 3, 612.

MILLER, A.B. & BULBROOK, R.D. (1980). The epidemiology and etiology of breast cancer. *N. Engl. J. Med.*, **303**, 1246.

MOOLGAVKAR, S.H., DAY, N.E. & STEVENS, R.G. (1980). Two-stage model for carcinogenesis: epidemiology of breast cancer in females. *J. Natl Cancer Inst.*, **65**, 1097.

MOORE, J.W., CLARK, G.M.G., HOARE, S.A. *et al.* (1986). Binding of oestradiol to blood proteins and aetiology of breast cancer. *Int. J. Cancer*, **38**, 625.

PIKE, M.C., ROSS, R.K., LOBO, R.A. *et al.* (1989). LHRH agonists and the prevention of breast and ovarian cancer. *Br. J. Cancer*, **60**, 142.

POWLES, T.J., FORD, H.T. & GAZET, J.C. (1987). A randomised clinical trial to compare tamoxifen with danazol for treatment of benign mammary dysplasia. *Senologia*, **2**, 1.

POWLES, T.J., HARDY, J.R. & ASHLEY, S.E. (1989). A pilot trial to evaluate the acute toxicity and feasibility of tamoxifen for prevention of breast cancer. *Br. J. Cancer*, **60**, 126.

ROSSNER, S. & WALLGREN, A. (1984). Serum lipoprotein after breast cancer surgery and effects of tamoxifen. *Atherosclerosis*, **52**, 339.

SAKAI, F., CHEIX, F., CLAVEL, M. *et al.* (1978). Increase in steroid binding globulin induced by tamoxifen in patients with breast cancer. *J. Endocrinol.*, **76**, 219.

SHERMAN, B.M., CHAPLER, F.K., CRICKARD, K. & WYCOFF, D. (1979). Endocrine consequences of continuous antioestrogen therapy with tamoxifen in premenopausal women. *J. Clin. Invest.*, **64**, 398.

STEWART, H.J. & KNIGHT, G.M. (1989). Tamoxifen and the uterus and endometrium. *Lancet*, **i**, 375.